# Professional Notes

**Published:** 1887-11-26

**Authors:** Geo. W. Potter


### Professional Notes.

By Geo. W. Potter, M.D.

Concerning the action of the epiglottis in swallowing, Mr. Carmalt Jones (*Journal of Laryngology*) says : "It used to be taught that the epiglottis had the action of a lid, and that one of its functions was in the act of swallowing to shut down over the larynx as if it were hinged on at the base of the tongue, and thus to prevent food, etc., from passing into the larynx, and that in addition, the epiglottis, being thus tilted downwards and backwards, acted as a sort of guide or shoot for the food, and directed it towards the upper end of the œsophagus when pushed backwards by the base of the tongue. I do not believe that the epiglottis has any such action. Occasionally it is seen with the laryngoscope hanging backwards, and so far covering the larynx that no view of the interior can be obtained, giving the idea that it would only have to shut down a little more to form a complete protection for the larynx against the entrance of foreign bodies, such as food, but also suggesting that in vomiting it would be just in the very position to intercept material on its passage upwards and forwards, and to direct it into the larynx. With the mirror I have often seen that the epiglottis has the power to *close its sides together*. The lateral borders approximate in the middle line, the front of the epiglottis remaining erect, and there is only a small round or oval opening behind the centre of the tip of the epiglottis. There are also cases where the epiglottis stands up very prominently behind the base of the tongue, and can be seen without the mirror ; by drawing the tongue forward it can then be seen to diminish its breadth by folding its sides together, but is not seen to flap backwards. One simple experiment can be tried by anyone. Take a little water in the mouth, protrude the tongue as far as possible, close the teeth on it, and then, with the tongue thus held, swallow the water. It will be found that the water is perfectly easily swallowed without much movement of the tongue, but that a great deal is done by the muscles of the pharynx. Taking together the result of this experiment, Kirke's statement that even when the epiglottis is destroyed there is little danger of food passing into the larynx so long as its muscles can act freely, and the knowledge that in many cases where the epiglottis can be seen by the laryngoscope to be clearly eroded, yet the patient can swallow solids and fluids without any signs of choking, I submit that in swallowing, the epiglottis is not carried downwards and backwards over the entrance to the larynx, but remains upright and closes its sides together."

---

According to L. H. Petit, a certain number of spontaneous hæmorrhages (epistaxis, hæmorrhoids, &c.) and secondary traumatisms owe their origin to the hepatic condition. Hepatic revulsion, by cold douches, or large blisters over the hepatic region, leads to the cessation of these hæmorrhages. Pachard, Sales, and Gerard Marchant have also made observations on the subject. Pachard reported the case of a lady with cirrhosis of the liver, in whom a severe epistaxis occurred which defied all the usual methods of arrest. A blister over the liver stopped it. Gerard Marchant gave the case of a man of sixty who was attacked with epistaxis and bleeding from the gums after intermittent fever in 1874, and again in 1876 and 1878. In 1874 two teeth were removed and cauterisation applied unsuccessfully, blood continuing to flow freely from the nose and mouth for no less than twelve days. This was finally arrested by sulphate of quinine. In May, 1887, abundant and repeated epistaxis occurred. Tampons failed entirely to check it. An enlarged liver was found, and Marchant applied a large blister over the hepatic region. The epistaxis was diminished in twenty minutes, and at the end of an hour was arrested, and has never recurred. These cases are well worth bearing in mind by those who are far from consultational help in outlying districts.

---

Speaking of health resorts for laryngeal and other forms of phthisis the *Journal of Laryngology* says : "Most of the German saline springs are situated on the range of the Taunus Hills. Of these, unquestionably one of the very best is Soden. Containing, as it does, some twenty-three springs, with a temperature varying between 12 deg. and 18 deg. C., and with one thermal spring of a temperature of 30 deg. C., the waters of which are charged with chloride of sodium in varying degrees up to 116 grammes per litre, and some of which are further charged with carbonic acid (notably the Champagnerbrunn), the health resort of Soden is, though at the present one of the less known of German spas, unquestionably one of the very best places for the treatment of pulmonary and catarrhal complaints. Additional advantages are the complete protection of the village from north winds, the comparatively high altitude and equable climate, which make it, of all others in Germany, the resort most suitable for the treatment of phthisis, either of throat or lungs, and catarrhal conditions generally. No spa possesses the peculiar and distinctive advantages of Soden. Though a small amount of iron is present in the waters, they are not on that account contra-indicated in the treatment of incipient phthisis. *Compressed pastilles* of Soden water are now an article of commerce, as well as the mineral waters. These are made from two of the well waters, and, containing a large amount of chloride of sodium, are particularly serviceable in pharyngeal catarrhs, and may even in some degree be used where it is desired to obtain the effect of the Soden treatment in persons who are unable to make the necessary journey to the spa itself." Having had a limited personal experience of these Soden pastilles, we are able to that extent to confirm this judgment of their value in pharyngeal catarrh. It may be added that they are quite safe, and very agreeable in taste.

---

Tracheotomy is a desperate remedy, and should only be had recourse to when all other methods of relief and cure have failed. *Intubation* is said to be preferable in many cases, and its advocates insist that it should always be tried before tracheotomy is decided upon. F. Huber publishes a study of forty-seven cases. He affirms that intubation has passed through its experimental stage. Of the forty-seven cases, twenty-nine, with eleven recoveries, were in children under three years' of age ; eighteen, with nine recoveries, in children of three years or over. Of the eleven children under three (who recovered), one was nine and a-half months old, one a year old ; two, two years ; two, two and a-half ; and two, two years and eight months old. Of all the children he had been called to treat for laryngeal stenosis, he had found it necessary to resort to intubation in only one out of every three or four. The operation should not be done until dangerous symptoms supervene. Huber employed it in exactly the same class of cases as he would have performed tracheotomy in, a year ago. In order to avoid accidents, the efforts to place the tube in position should be short and repeated, rather than kept up continuously and uninterruptedly for any length of time. In a case in which the dislodged membrane was pushed downwards before the tube, brandy was given, which excited cough, and caused the expulsion both of the membrane and the tube. In favourable cases the tube may be removed as early as the fourth or fifth day, although it is often desirable to leave it longer. It may usually be dispensed with at a much earlier date than the tracheotomy tube. It is as a rule advisable to leave the tube in until all traces of diphtheritic patches have disappeared from the pharynx. Intubation may be practised at any age.

